# Supplementary material for: Accelerating discovery across scientific disciplines through reproducible workflows with AiiDAlab
Source: Digit Discov. 2026 Apr 22;5(5):2310–24. doi: 10.1039/d5dd00567a (PMC13158844; doi:10.1039/d5dd00567a)
Supplement: DD-005-D5DD00567A-s001 [file DD-005-D5DD00567A-s001.pdf]

Supplementary Information (SI) for Digital Discovery.
This journal is © The Royal Society of Chemistry 2026

Supplementary Information for: Accelerating discovery across scientific disciplines through reproducible workflows with AiiDAlab

Aliaksandr V.Yakutovich,[a,b,†] Daniel Hollas,[c,†] Edan Bainglass,[d,†] Jusong Yu,[d,†] Corsin Battaglia,[b,e,f] Miki Bonacci,[d] Lucas Fernandez Vilanova,[g] Stephan Henne,[g] Anders Kaestner,[h] Michel Kenzelmann,[h] Graham Kimbell,[b] Jakob Lass,[h] Fabio Lopes,[a] Daniel G. Mazzone,[h] Andres Ortega-Guerrero,[a] Xing Wang,[*d] Nicola Marzari,[d,i] Carlo A. Pignedoli,[*a] and Giovanni Pizzi,[*d,i]

1 Introduction

The following supplementary information includes further details on AiiDAlab-experiment integration and handling of large data, as well as more technical details regarding deploying and managing AiiDAlab servers (cloud and local). We also discuss "under-the-hood" developments taken to improve the performance of the platform and the experience of its users.

2 AiiDAlab for PSI's ICON beamline

We noted in the main manuscript the applicability of the approach taken with CAMEA also for neutron imaging experiments at the PSI ICON beamline[1]. In this case, the information registered in neutron imaging experiments is mainly composed of 2D images, showing the attenuation of the incident neutron beam caused by the interaction with the scanned sample. The amount of data produced during an experiment easily reaches tens of GBs, but hundreds of GBs are not rare: this introduces a bottleneck for the analysis when data must be copied between storage locations. The availability of a central collection of reduction and analysis workflows acting directly on the central storage would speed up the work during and after experiment. The images are used in different contexts, resulting in a series of images that are processed differently depending on the scanning scheme: it can be time-series in 2D observing processes in the sample, tomography scans that can be reconstructed into 3D volumes showing the distribution of attenuation coefficients, wavelength scans to observe diffraction patterns in crystalline materials, and interferometer scans to probe correlation lengths in the sample. These techniques can also be combined, increasing the analysis's complexity. Typical workflows are divided into experiment setup, reduction, and analysis. Basic inspection and tuning tasks that require fluent user interaction are performed during the experiment setup. Today, this is often done using tools like ImageJ[2], which has turned into a *de facto* standard for imaging. There is potential to integrate some of these tasks, like determining resolution and pixel size, into AiiDAlab to benefit from the documented workflow. The reduction involves operations that can be easily generalized into efficient user tools. Tomographic reconstruction is a task that can be partly integrated, in particular, to perform multiple reconstructions using the same parameters. The tuning is, however, more convenient using existing GUI applications like MuhRec[3]. Conversely, the analysis is often more complex to generalize, as it depends much on the investigated sample and process and the experiment objectives. The analysis mostly requires a toolbox of image processing operations rather than predefined interactive tools. Data visualization is a further task that is common for imaging applications. Here, we see some potential in basic rendering to provide an impression of the data using, for example, PyVista[4], which integrates well with Jupyter notebooks. Finally, when focusing on supporting advanced visualization, we envision preparing the data in ways that make using existing open-source and commercial tools easier, e.g., applying advanced denoising filters and storing the data using efficient data formats.

3 Large data sets

In materials-science simulations, it is common to store 3D data (such as Hartree potentials, electron densities, or orbitals) in Cube files (https://paulbourke.net/dataformats/cube/), which can easily reach several gigabytes in size. However, direct visualization in a browser-based environment is often impractical and might not be feasible due to bandwidth and browser limitations.

[a] *nanotech@surfaces Laboratory, Empa-Swiss Federal Laboratories for Materials Science and Technology, 8600 Dübendorf, Switzerland*
[b] *Materials for Energy Conversion Laboratory, Empa-Swiss Federal Laboratories for Materials Science and Technology, Dübendorf, CH-8600, Switzerland*
[c] *Centre for Computational Chemistry, School of Chemistry, University of Bristol, BS8 1TS Bristol, UK*
[d] *PSI Center for Scientific Computing, Theory and Data, Paul Scherrer Institute, 5232 Villigen PSI, Switzerland*
[e] *Department of Information Technology and Electrical Engineering, ETH Zurich, 8092 Zurich, Switzerland*
[f] *Institute of Materials, School of Engineering, École Polytechnique Fédérale de Lausanne, 1015 Lausanne, Switzerland*
[g] *Laboratory for Air Quality / Environmental Technology, Empa-Swiss Federal Laboratories for Materials Science and Technology, 8600 Dübendorf, Switzerland*
[h] *PSI Center for Neutron and Muon Sciences, Paul Scherrer Institute, 5232 Villigen PSI, Switzerland*
[i] *Theory and Simulation of Materials (THEOS), and National Centre for Computational Design and Discovery of Novel Materials (MARVEL), École Polytechnique Fédérale de Lausanne, 1015 Lausanne, Switzerland*
† These authors contributed equally to the work.
∗ Corresponding authors e-mails: xing.wang@psi.ch, carlo.pignedoli@empa.ch, giovanni.pizzi@psi.ch.

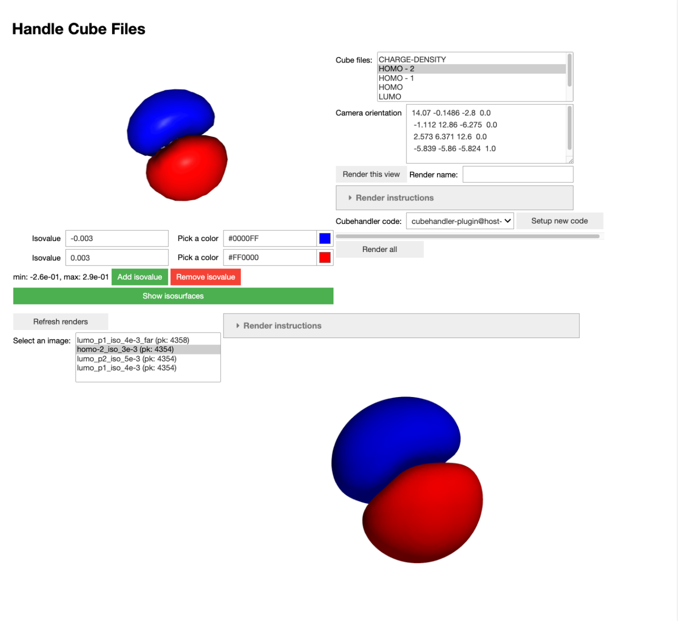

Fig. 1 Top: An interface to visualize low-resolution volumetric data files (in this case, a "Gaussian cube file" for electronic orbitals) and request their high-resolution rendering. Bottom: The high-resolution rendered cube file.

We addressed this challenge by developing an alternative strategy that allows users to handle large volumetric data indirectly and more efficiently in the **cubehandler** tool[5]. Among other functionalities, the tool enables effective data reduction, shrinking files by up to two orders of magnitude. Shrinkage is usually applied directly within computational workflows that produce Cube files as output, and is achieved by both reducing the grid density on which properties are stored, and using a smart truncated format for the data stored in the cube file, avoiding unnecessarily storing numbers with too many significant digits. The resulting reduced files, often just a few megabytes in size, are then downloaded and stored in AiiDAlab for rapid inspection. Importantly, the relationship between the original and shrunk files is preserved in the AiiDA database. If, after reviewing the reduced version, a user wishes to generate a high-quality image based on the full-resolution data, they can launch a follow-up rendering job (see Fig. 1). This job automatically uses the original Cube file and is configured according to the visualization settings (such as camera orientation) selected during the preview.

More generally, data movement is an essential task when working with large datasets, particularly in domains such as climate modeling (see main manuscript), where simulations can sometimes produce terabytes of data. Indeed, at large supercomputing centers, simulations typically are required to write files in scratch file systems, temporary storage areas where files are automatically deleted after a period of inactivity (typically a few weeks). This makes it critical to implement efficient data handling, as large volumes of data must be moved to the scratch space before the simulation, and then to long-term storage afterwards. In these scenarios, we leverage AiiDA's built-in data stashing capabilities. Once a simulation completes, AiiDA can automatically transfer selected output files to a predefined and more permanent storage location directly on the remote server, ensuring both that the data movement is efficient, and that the data remains accessible for future use without transferring it to the AiiDAlab server (that might have limited storage).

It is worth noting that both visualization and remote data management are highly domain-specific challenges. Visualization is inherently tied to the nature of the data: different types of data require different representation methods. Similarly, remote storage and data stashing depend heavily on the underlying storage technology, whether it is a traditional hierarchical file system, a tape archive, or an object store. These differences necessitate tailored solutions for each use case, both in terms of how data is presented and how they are stored and retrieved.

4 AiiDAlab deployments

4.1 AiiDAlab demo server

The demo server is accessible to all registered GitHub users at `https://demo.aiidalab.io`. Once logged in, new users can begin running calculations immediately. The current version of the demo server features a ready-to-use AiiDAlab Quantum ESPRESSO (QE) app[6]. Although the server offers limited computational resources, users can run simple illustrative examples. All necessary components, including compiled executables from the Quantum ESPRESSO suite[7,8], the pseudopotential files required to run the simulations, and the AiiDA workflows to drive the simulations, are pre-installed and ready for use.

It is essential to note that the demo server is not intended for production use, due to the significant financial cost associated with such a general service. Therefore, storage is deleted 12 hours after login. On the other hand, we invested significant effort in ensuring that the experience of users logging in for the first time is as simple as possible, allowing them to submit their first simulation in a matter of minutes (and usually starting their AiiDAlab instance in less than a minute). This is achieved by pre-installing the Quantum EPSRESSO app and all its dependencies (e.g., the Quantum ESPRESSO code, the pseudopotential libraries, a pre-configuring AiiDA database) in the base AiiDAlab image - a blueprint used by Kubernetes when deploying AiiDAlab instances.

4.2 Local deployments

When a quick and straightforward setup is needed to run AiiDAlab for a single user on their computer, we recommend to the user the **aiidalab-launch** tool. This command-line utility, developed by the AiiDAlab team and powered by Docker, enables users to deploy AiiDAlab locally with minimal effort. It allows for creating and managing multiple profiles, each customizable with specific Docker images, port numbers, and volume mappings. The utility uses Docker in the background to generate AiiDAlab containers from the specified (or default) AiiDAlab image. With simple commands for starting, stopping, and monitoring instances, and built-in support for automatic browser launching and port forwarding, **aiidalab-launch** eliminates the complexities of container orchestration, making advanced computational workflows

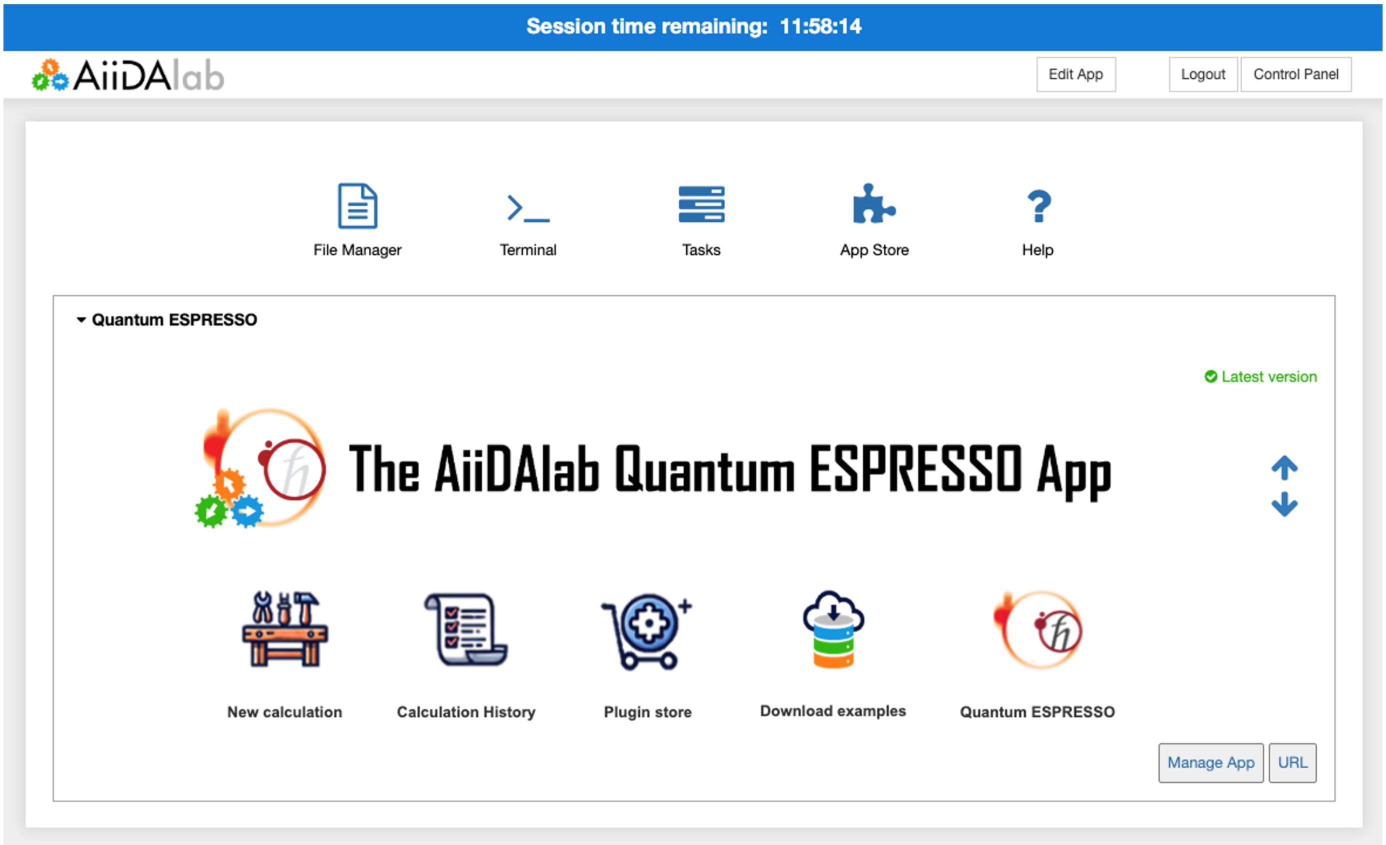

Fig. 2 AiiDAlab demo server: the user is presented with an instance that is fully set up and ready to run Quantum ESPRESSO simulations. All installations are pre-executed when preparing the Docker image of the server, so that we can reduce the time from login to first user action to below one minute.

more accessible. For further information, we suggest visiting the tool's GitHub page and reading about its requirements and usage[9].

4.3 Managing an AiiDAlab server

While the **aiidalab-launch** solution is ideal for individual users or single-machine deployments, for use cases that require multi-user access (e.g., research groups, organizations, institutions), a scalable Kubernetes-based setup[10] is more appropriate. In this setup, each user accesses a containerized environment fully isolated from other users, i.e., data remain private to each user. Moreover, this setup allows system administrators to access user sessions for support and troubleshooting, improving the overall user experience. While the configuration and maintenance of Kubernetes deployments require some expertise, recent updates have simplified this deployment strategy. Deploying AiiDAlab in this context involves using the standard JupyterHub Helm chart, a blueprint bundle used by Kubernetes to deploy JupyterHub[11]. Additionally, the Kubernetes ecosystem provides a comprehensive set of tools for backing up, monitoring, and administering multi-user environments.

For medium-sized deployments (less than ∼50 users) that do not require scaling on multiple computer nodes, we offer a deployment guide for using Micro-Kubernetes (MicroK8s), which simplifies Kubernetes management on a single server (either a physical or virtual machine). For larger deployments with more than 50 users, a full Kubernetes cluster setup is recommended to ensure scalability and high performance. This broad range of deployment options that we provide offers great flexibility, enabling AiiDAlab to scale according to user needs, from single users to small research groups and larger institutional setups. For more details on the latest features and deployment guides for MicroK8s and Kubernetes, refer to AiiDAlab's official repositories and documentation[12].

It is worth noting that users can begin with a single-user deployment and later migrate to a Kubernetes cluster (or vice versa) without losing any of their generated data. Both options rely on the same AiiDAlab image, which is built using the recipes provided in the **aiidalab-docker-stack**[13] repository. This image is based on the official **jupyter/minimal-notebook** image[14], ensuring compatibility with the standard Jupyter infrastructure while providing all the necessary components to run AiiDAlab.

5 "Under-the-hood" improvements

Enhancements such as new features, apps, and widgets are usually easy to notice since they directly impact the user experience through GUI modifications. However, for a service like AiiDAlab to be truly successful, equal attention must be paid to less visible but equally important aspects.

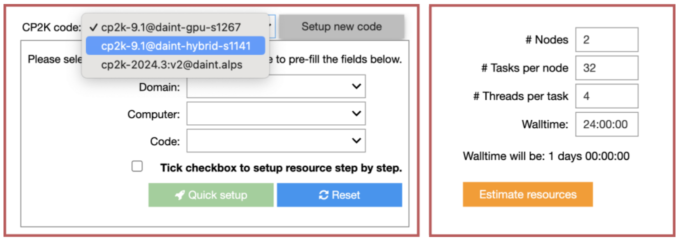

Fig. 3 (left) Widget to set up the computer/code to run the simulations. Once a suitable computer/code combination is chosen, users can click "Quick setup" to complete the configuration. Users may also modify the setup details for advanced use cases, although this requires a deeper understanding of the underlying computational infrastructure. (Right) Resource estimation widget, that helps users in selecting an appropriate number of computational resources and parallelization strategy. While the widget is general, the logic needs to be implemented for each code. Therefore, at the moment this widget is available only for some selected apps, such as those using the CP2K code.

One such aspect is the stability of the service, where we have invested significant development efforts. For example, core services are now automatically tested before each release and deployment in the production environment. Another key factor is the quality of the code, which we continuously improve by fixing bugs and refining implementations.

We also focus on ensuring secure and reliable access to HPC resources, which has grown more complex as security requirements evolve. At the Swiss National Supercomputing Centre (CSCS), for example, access once relied on standard SSH key pairs. Since, however, mandatory Multi-Factor Authentication (MFA) was introduced, requiring daily re-authentication. While this improves security, it complicates automated workflows. To address this, we developed the **aiidalab-mfa-cscs** app [15], which helps users securely manage SSH keys. In parallel, CSCS is deploying modern solutions such as the FirecREST API [16,17], for which native support in AiiDA is provided via the **aiida-firecrest** [18] plugin.

On the GUI front, AiiDAlab now offers improved structural visualization and manipulation features. Users can apply distinct visual representations, such as ball-and-stick models, to selected atoms, enabling more detailed and customized views of atomistic structures. Additionally, the ability to directly edit cell vectors and perform transformations using a transformation matrix has been implemented. The enhanced widgets support structures with varying periodicities, allowing users to explore materials ranging from non-periodic molecules to fully periodic crystals.

AiiDAlab also includes a template-based system for setting up computers and codes with a dedicated widget to configure computational resources (Fig. 3 left). Users can select from preconfigured codes or set up a new one from scratch. The widget interfaces with the AiiDA resource registry [19], which stores information about various known computational environments.

Lastly, to ensure a smooth and responsive user experience, modern frontend techniques (e.g., UI lazy-loading) have already been implemented in the Quantum ESPRESSO app and are actively being promoted to other app developers. These approaches leverage well-established design patterns (e.g., Model-View-Controller, Observer, and Mediator) to decouple the app's data models from its UI components, significantly improving the responsiveness of AiiDAlab applications.

Notes and references

1 A. Kaestner, S. Hartmann, G. Kühne, G. Frei, C. Grünzweig, L. Josic, F. Schmid and E. Lehmann, *Nucl. Instrum. Methods Phys. Res. A*, 2011, **659**, 387–393.

2 C. A. Schneider, W. S. Rasband and K. W. Eliceiri, *Nat. Methods*, 2012, **9**, 671–675.

3 A. P. Kaestner, *Nucl. Instrum. Methods Phys. Res. A*, 2011, **651**, 156–160.

4 B. Sullivan and A. Kaszynski, . *Open Source Softw.*, 2019, **4**, 1450.

5 Empa nanotech@surfaces laboratory, *cubehandler*, GitHub repository: `https://github.com/nanotech-empa/cubehandler`, 2025, Accessed: 2025-12-08.

6 X. Wang, E. Bainglass, M. Bonacci, A. Ortega-Guerrero, L. Bastonero, M. Bercx, P. Bonfà, R. De Renzi, D. Du, P. N. O. Gillespie, M. A. Hernández-Bertrán, D. Hollas, S. P. Huber, E. Molinari, I. J. Onuorah, N. Paulish, D. Prezzi, J. Qiao, T. Reents, C. J. Sewell, I. Timrov, A. V. Yakutovich, J. Yu, N. Marzari, C. A. Pignedoli and G. Pizzi, *npj Computational Materials*, 2026, **12**, 72.

7 P. Giannozzi, S. Baroni, N. Bonini, M. Calandra, R. Car, C. Cavazzoni, Davide Ceresoli, G. L. Chiarotti, M. Cococcioni, I. Dabo, A. D. Corso, S. d. Gironcoli, S. Fabris, G. Fratesi, R. Gebauer, U. Gerstmann, C. Gougoussis, Anton Kokalj, M. Lazzeri, L. Martin-Samos, N. Marzari, F. Mauri, R. Mazzarello, Stefano Paolini, A. Pasquarello, L. Paulatto, C. Sbraccia, S. Scandolo, G. Sclauzero, A. P. Seitsonen, A. Smogunov, P. Umari and R. M. Wentzcovitch, *J. Phys. Condens. Matter*, 2009, **21**, 395502.

8 P. Giannozzi, O. Andreussi, T. Brumme, O. Bunau, M. B. Nardelli, M. Calandra, R. Car, C. Cavazzoni, D Ceresoli, M. Cococcioni, N. Colonna, I. Carnimeo, A. D. Corso, S. d. Gironcoli, P. Delugas, R. A. D. Jr, A Ferretti, A. Floris, G. Fratesi, G. Fugallo, R. Gebauer, U. Gerstmann, F. Giustino, T. Gorni, J. Jia, M. Kawamura, H-Y Ko, A. Kokalj, E. Küçükbenli, M. Lazzeri, M. Marsili, N. Marzari, F. Mauri, N. L. Nguyen, H.-V. Nguyen, A Otero-de-la-Roza, L. Paulatto, S. Poncé, D. Rocca, R. Sabatini, B. Santra, M. Schlipf, A. P. Seitsonen, A. Smogunov, I Timrov, T. Thonhauser, P. Umari, N. Vast, X. Wu and S. Baroni, *J. Phys. Condens. Matter*, 2017, **29**, 465901.

9 AiiDAlab Team, *AiiDAlab Launch*, GitHub repository: `https://github.com/aiidalab/aiidalab-launch`, 2025, Accessed: 2025-12-08.

10 The Kubernetes Authors, *Kubernetes website*, `https://kubernetes.io/`.

11 Jupyter, *Jupyterhub Helm charts*, `https://github.com/jupyterhub/helm-chart`.

12 AiiDAlab Team, *AiiDAlab Deployment Guide*, `https://aiidalab.readthedocs.io/en/latest/admin/index.html`, 2025, Accessed: 2025-12-08.

13 AiiDAlab Team, *Docker Stack for AiiDAlab*, GitHub repository:

https://github.com/aiidalab/aiidalab-docker-stack, 2025, Accessed: 2025-12-08.

14 *Jupyter Docker Stacks*, GitHub repository: https://github.com/jupyter/docker-stacks, 2025, Accessed: 2025-12-08.

15 AiiDAlab Team, *aiidalab-mfa-cscs*, GitHub repository: https://github.com/aiidalab/aiidalab-mfa-cscs, 2025, Accessed: 2025-12-08.

16 CSCS, *firecrest*, GitHub repository: https://github.com/eth-cscs/firecrest, 2025, Accessed: 2025-12-08.

17 E. Palme, J. P. Dorsch, A. Khosravi, G. Pizzi, F. Pagnamenta, A. Ceriani, E. Koutsaniti, R. Sarmiento, I. Bonesana and A. Dabin, *FirecREST v2: lessons learned from redesigning an API for scalable HPC resource access*, 2025, https://arxiv.org/abs/2512.11634.

18 AiiDA Team, *aiida-firecrest*, GitHub repository: https://github.com/aiidateam/aiida-firecrest.

19 AiiDA Team, *AiiDA Resource registry*, GitHub repository: https://github.com/aiidateam/aiida-resource-registry, 2025, Accessed: 2025-12-08.
